# Reply to Peters and Pittet, “Influenza and Alcohol-Based Handrub: the Danger of Ignoring Clinical Relevance,” and Boyce, “Alcohol-Based Handrubs and Influenza A”

**DOI:** 10.1128/mSphere.00745-19

**Published:** 2019-11-27

**Authors:** Ryohei Hirose, Takaaki Nakaya

**Affiliations:** a Department of Molecular Gastroenterology and Hepatology, Graduate School of Medical Science, Kyoto Prefectural University of Medicine, Kyoto, Japan; b Department of Infectious Diseases, Graduate School of Medical Science, Kyoto Prefectural University of Medicine, Kyoto, Japan

## REPLY

We thank Drs. Peters and Pittet and also Dr. Boyce for their comments on our article (1, 2). First, we wish to clarify that the objective of our study was to perform basic research rather than to emphasize immediate clinical relevance. Our conclusion was not intended to encourage changes in current infection prevention and control (IPC) practices. Instead, our aim was to provide *in vitro* data that could lead to improvements in IPC practices in the future. We do not doubt that existing IPC practices are currently the most effective and should be followed by health care workers to ensure patient safety (3).

We recognize that our *in vivo* disinfectant evaluations (i.e., finger pad tests modified for this study) may not reflect the situations in clinical practice. It is very difficult to replicate the convection caused by hand rubbing or tube inversion exactly in each experimental condition. Therefore, tube inversion or hand rubbing were intentionally not included in these disinfectant evaluations to examine the effectiveness of ethanol on influenza A virus (IAV) in saline and mucus (4). Hand rubbing is expected to contribute to an increase in ethanol concentration in infectious mucus owing to an increase in the level of mixing between ethanol and mucus by convection. As actual antiseptic hand rubbing (AHR) involves actively rubbing the hands, the effect of AHR on infectious mucus is assumed to be higher than that observed in our study. Therefore, based on our findings, we cannot conclude that AHR practiced clinically is less effective against infectious mucus.

In contrast, as the degree (strength) of hand rubbing is difficult to evaluate objectively, it has not yet been demonstrated that all surfaces of hands are rubbed together with enough strength to inactivate infectious mucus. If there is an area on the hand surface that is not sufficiently rubbed, the disinfectant effect on infectious mucus adhering to this area is expected to be close to that observed in our study. We have also developed a system that can objectively evaluate the degree (strength) of hand rubbing, and we are examining the scientific significance of the act of hand rubbing and the effects of handrubs on disinfectant effectiveness on infectious mucus.

Next, in this study, samples that were positive for IAV were selected from completely anonymized sputum samples. As these did not include patients <20 years old and was not evaluated for coinfection, as Dr. Boyce points out, the samples may not have represented the majority of individuals with IAV, which was the limitation of this study. Nevertheless, in our preliminary study, the viscosities of all sputum samples (regardless of influenza positive or negative) were not very different but were about 10 to 100 times higher than that of saline. This result suggests that the rate of increase of ethanol concentration in mucus samples was lower than that in saline. Therefore, we assume that the results of *in vitro* inactivation tests will not change significantly.

This study used pure ethanol with no additional ingredients to better match the setting conditions of fluid simulation of the changes in ethanol concentrations in infectious mucus. As additional ingredients can affect the results, it is necessary to confirm the effect of additional ingredients. We plan to conduct evaluations using commercially available alcohol-based handrub products in the future.

Not all of the factors potentially influencing disinfection effectiveness have been identified. Our study revealed that the viscosity of infectious body fluids can greatly influence disinfectant effectiveness, and we think it is necessary to continue evaluating the potential role of viscosity and other unexplored characteristics of body fluids in the effectiveness of current hand hygiene protocols.
